# Leptomeningeal metastasis from lung adenocarcinoma associated with Lemierre syndrome in a middle-aged man: a case report and review of the literature

**DOI:** 10.1186/s12879-025-12359-3

**Published:** 2026-01-14

**Authors:** Xiaoxia Yang, Jingang Han, Zhi Yu, Jianhua Nian, Jie Chen

**Affiliations:** Department of Vascular Surgery, The Second Affiliated Hospital of Zhejiang Chinese Medical University, Hangzhou, China

**Keywords:** Lemierre syndrome, Adenocarcinoma of lung, Leptomeningeal metastasis, *Fusobacterium necrophorum*, Tyrosine kinase inhibitors, Multidisciplinary management

## Abstract

**Background:**

Lemierre syndrome (LS), predominantly triggered by oropharyngeal infection with *Fusobacterium necrophorum*, is a rare but life-threatening condition. The co-occurrence of LS with leptomeningeal metastases (LM) from lung adenocarcinoma (LADC) is exceptionally scarce, with only a handful of cases documented in the literature.

**Case presentation:**

A 42-year-old male presented with neck pain and infection, which progressed to cervical venous thrombosis, cervical lymphadenopathy, and multiple pulmonary nodules, features consistent with a diagnosis of LS. The patient received targeted treatment for LS, including anticoagulation, broad-spectrum antibiotics with anaerobic coverage (piperacillin-tazobactam 4.5 g every 8 h for 2 weeks), and anti-inflammatory agents. Shortly after the successful LS treatment, the patient developed chest pain; computed tomography (CT) imaging revealed residual lung nodules, and further pathological examination confirmed LADC. He subsequently started LADC-specific therapy combining targeted agents and chemotherapy. Later, the patient reported persistent headache; enhanced brain CT confirmed LM from LADC. His treatment regimen was adjusted to include an increased dose of osimertinib.

**Conclusions:**

This case underscores the importance of heightened clinicians’ awareness of the complex interplay between LADC leptomeningeal metastases and LS. Early recognition and multidisciplinary intervention in such multifaceted cases are crucial for optimizing patient outcomes.

**Clinical trial number:**

Not applicable.

**Supplementary Information:**

The online version contains supplementary material available at 10.1186/s12879-025-12359-3.

## Introduction

Lemierre syndrome (LS), often termed the “forgotten disease” due to its low incidence and potential for misdiagnosis, affects approximately 0.6–2.3 individuals per million annually [1]. Clinically, LS is defined by a triad: recent oropharyngeal infection, radiological or clinical evidence of internal jugular vein thrombosis, and isolation of anaerobic pathogens [2]. Specifically, *Fusobacterium necrophorum* accounts for over 80% of causative agents [3]. First documented in *The Lancet* in 1936 by French microbiologist André Lemierre [4], LS carries a high risk of severe systemic complications (e.g., pulmonary septic emboli and distant abscesses), and death if diagnosis or targeted treatment is delayed [3, 5]. Recent reviews highlight misdiagnosis of LS remains common, particularly in patients with underlying comorbidities that mask its classic manifestations, underscoring the need for heightened clinical vigilance in complex cases [6].

Lung cancer (LC) is a significant global health concern, representing a leading cause of cancer-related mortality worldwide [7]. Lung adenocarcinoma, the most prevalent histological subtype of LC, is a primary focus of this global health challenge. The metastatic potential of LADC is substantial. Crucially, LM is a major clinical challenge, with approximately 30% − 40% of patients with advanced LADC develop LM during disease progression [8]. In addition, approximately 30% − 40% of lung cancer patients experience bone metastasis during the course of their illness [9]. LM involves complex steps, including local invasion, intravasation, tumor cell circulation into the blood, blood-brain barrier disruption, tumor cell extravasation into the brain parenchymaand interaction with cells in the brain microenvironment [10–11].

Notably, the co-occurrence of LS and LADC-related LM is extremely rare. To our knowledge, this is the first reported case of LS occurring concurrently with EGFR-mutant LADC that progressed to LM. Given the clinical complexity of this dual condition, this case offers critical insights into diagnostic decision-making and treatment optimization. The purpose of this report is to detail the clinical course, diagnostic workup, and therapeutic adjustments for this patient, with the aim of improving clinician awareness of this rare comorbidity.

## Case report

The patient’s clinical presentation evolved significantly over five years, from LS to metastatic LADC and subsequently to LM. The corresponding timeline of diagnoses and management is detailed in Table 1.

**Table 1:** Case report timeline

Time	Diagnosis	Investigation	Management
July 1, 2020	42-year-old male presented with acute right-sided chest pain	Chest CT: Bilateral pulmonary inflammation, multiple scattered nodules/micronodules and mediastinal lymphadenopathy	suggestive of septic emboli
July 13, 2020	Definite diagnosis of LS	Neck Enhanced MRI: Right internal/external jugular & subclavian vein thrombosis confirmed.	LS treatment (anticoagulation, broad-spectrum antibiotics, anti-inflammatory agents) initiated
During Follow-up	Developed chest pain; Diagnosis of LADC confirmed	Pathology: Confirmed LADC.	LADC-specific therapy started, combining targeted agents and chemotherapy
2024–2025	Reported persistent headache; Diagnosis of LM confirmed	Brain Enhanced CT: LADC leptomeningeal metastasis confirmed. .	Treatment intensified with dose adjustment of the EGFR TKI, Osimertinib

### Stage 1: initial presentation

**Fig. 1:**
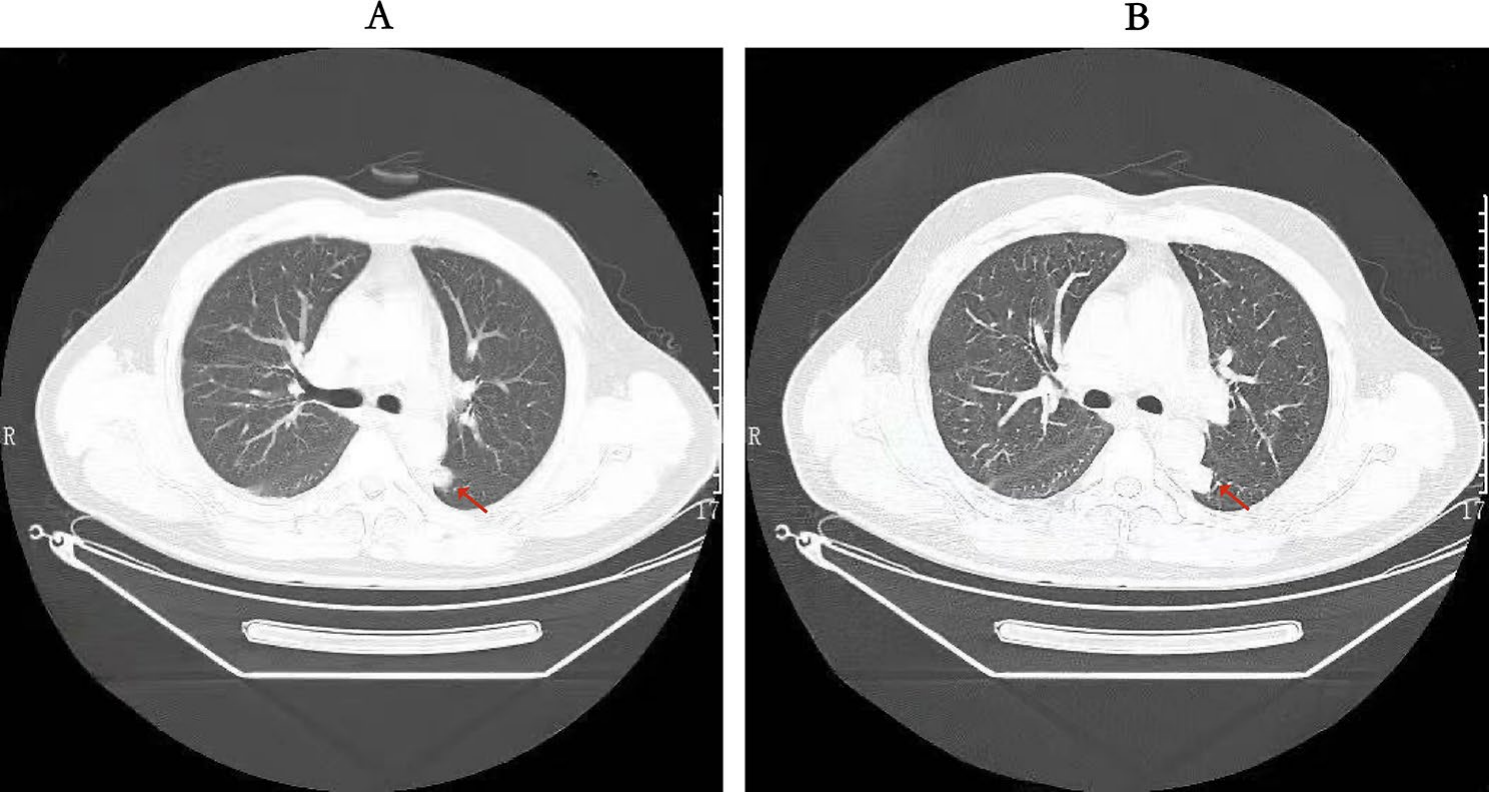
Chest CT scan on July 1, 2020. (**A**) and (**B**) are selected axial images demonstrating bilateral pulmonary inflammation, multiple scattered nodules and micronodules, and mild right pleural reaction, with arrows highlighting representative areas of consolidation and a dominant nodule

On July 1, 2020, A 42-year-old male with no significant medical history presented to the outpatient clinic, reporting sudden-onset right-sided pleuritic chest pain (worsening when lying down and alleviating upon changing to a semi-sitting or left lateral decubitus position). He denied fever, cough, dyspnea, nausea, or vomiting. During the outpatient visit, the patient underwent a fasting blood glucose test with a timing and result of 6:00 am, blood glucose 9.3 mmol/L. Glycated hemoglobin (HbA1c) test data: HbA1c 6.5%, HbA1 8.5%. The patient self-reported no history of smoking. A chest computed tomography (CT) performed on the same day revealed bilateral pulmonary inflammation, multiple scattered nodules and micronodules, bilateral localized pleural thickening, mild right lower lung pleural reaction (Fig. 1), and enlarged mediastinal lymph nodes; the patient was admitted for further workup.

Admission physical examination was notable only for coarse bilateral lung breath sounds. Laboratory tests (July 2) showed: normal platelet count (170 × 10^9^/L) and white blood cell count (8.3 × 10^9^/L), mildly elevated neutrophil percentage (63.5%), significantly increased C-reactive protein (CRP, 54.3 mg/L), D-dimer (4.83 mg/L), and fibrinogen (5.62 g/L), mild hyperglycemia (6.85 mmol/L), normal serum creatinine (58.9 µmol/L) and procalcitonin (0.05 ng/ml), and normal tumor markers (alpha-fetoprotein [AFP] 4.2 ng/ml, carcinoembryonic antigen [CEA] 1.4 ng/ml).

### Stage 2: diagnosis of Lemierre syndrome

**Fig. 2:**
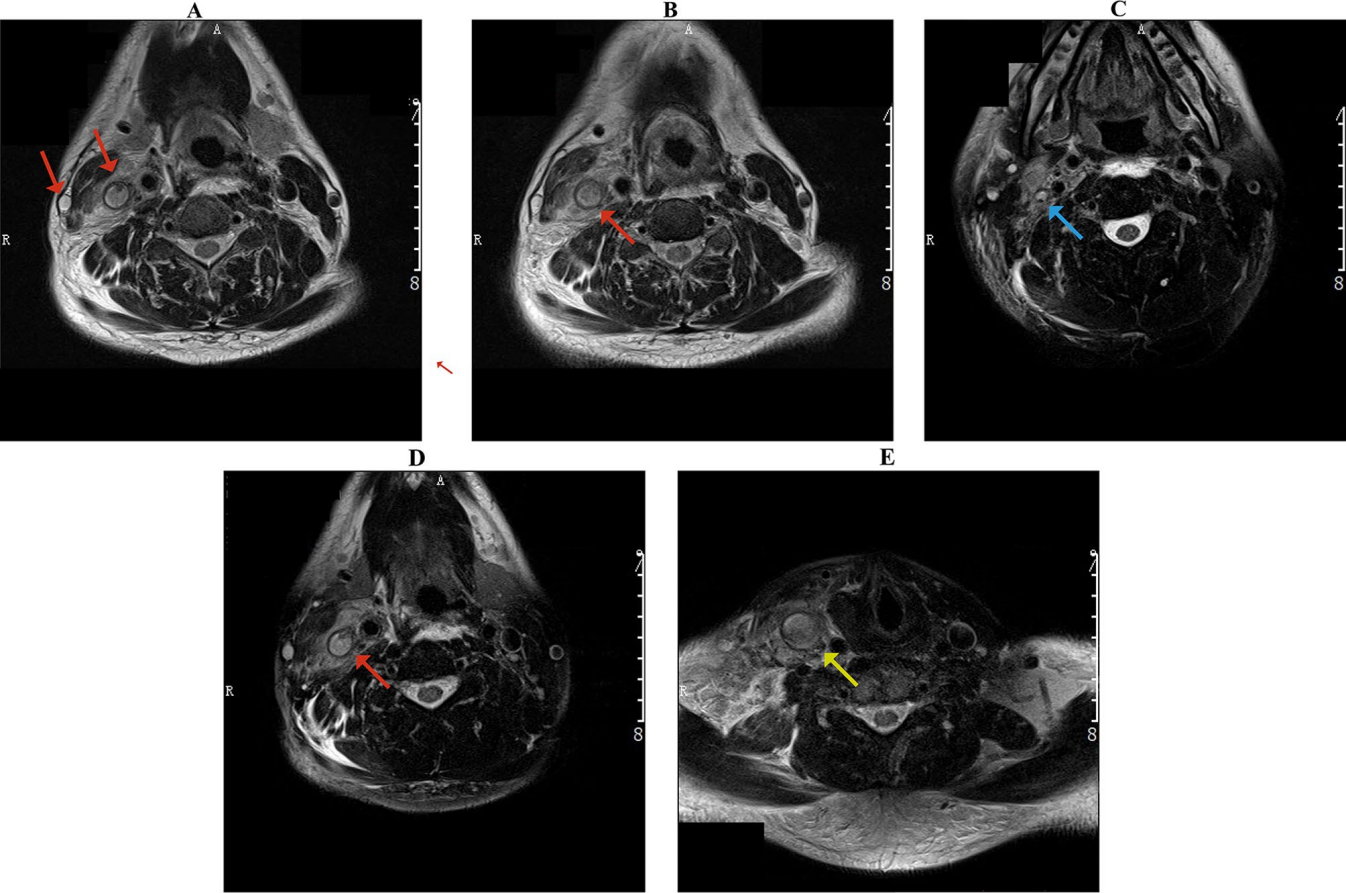
Cervical contrast-enhanced MRI (July 13, 2020). Axial images (**A**-**E**) show complex lesions: thrombosis of the right internal jugular, external jugular, and subclavian veins (**A**, **B**, **D**; red arrows), inflammatory changes in the right parapharyngeal space (**C**; red arrow), and multiple enlarged lymph nodes with clavicle osteomyelitis in the right supraclavicular fossa (**E**; red arrow)

During hospitalization, the patient developed swelling and pain in the right-side of the neck, accompanied by cervical lymphadenopathy, dysphagia (difficulty swallowing), and localized tenderness in the right anterior cervical region. Routine magnetic resonance imaging (MRI) performed on July 13 revealed thrombosis in the right internal and external jugular veins (Fig. 2), as well as the right subclavian vein. Inflammatory changes were noted in the right parapharyngeal space and supraclavicular fossa, along with multiple enlarged lymph nodes. Follow-up CT and MRI scans demonstrated stable pulmonary nodules and persistent pleural effusion with minimal absorption. Hepatic dysfunction and fatty liver were also noted (Fig. 2).

**Fig. 3:**
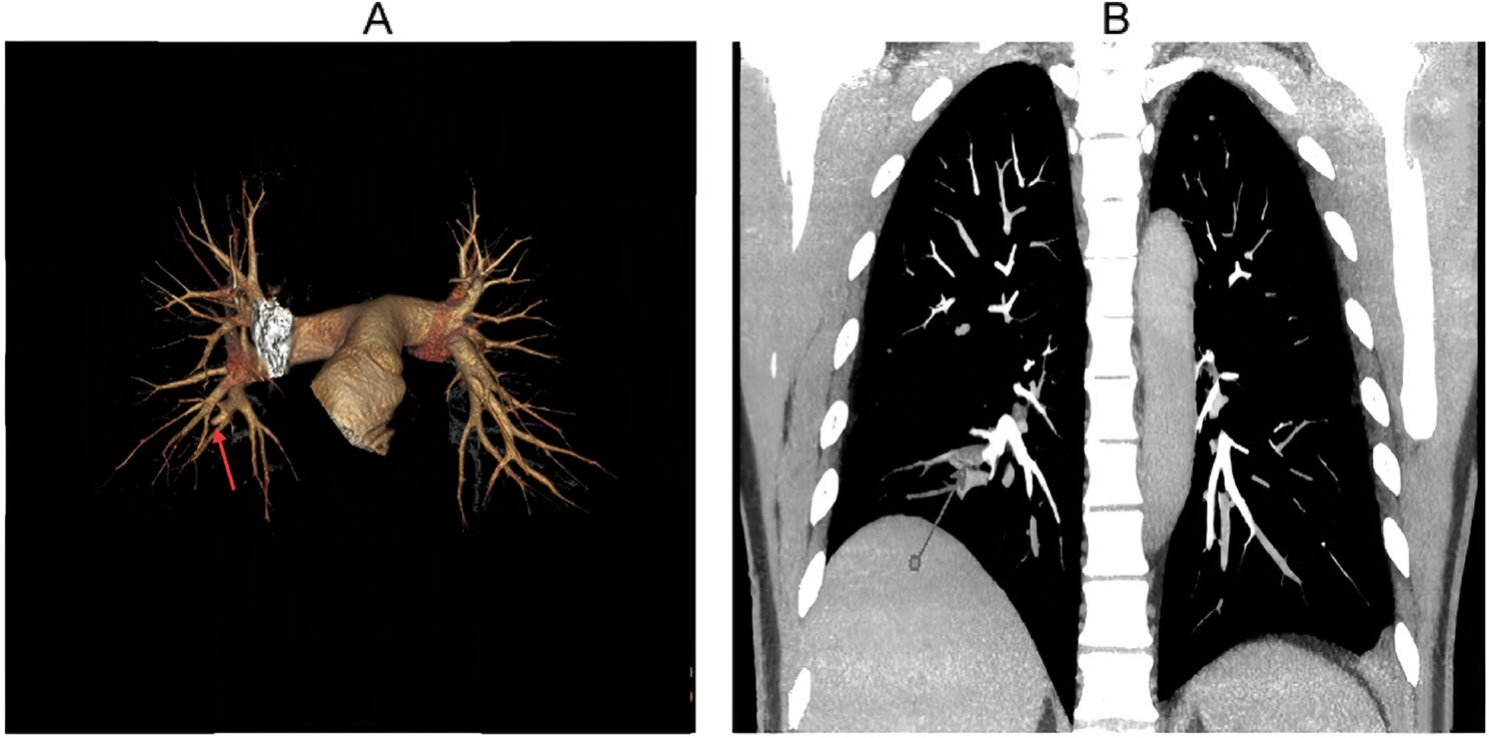
Computed tomography pulmonary angiogram (CTPA), July 17, 2020. (**A**) Segmental artery filling defect in the right lower lobe (red arrow), consistent with acute pulmonary embolism. (**B**) Bilateral scattered pulmonary nodules and micronodules, with the largest in the left upper lobe

A computed tomography pulmonary angiogram (CTPA) on July 17 (Fig. 3) detected right lower lung pulmonary arterial thrombosis, consistent with septic emboli. The CTPA also showed partial absorption of the bilateral pulmonary inflammation, while persistent pulmonary nodules/micronodules and left pleural effusion were noted.

**Fig. 4:**
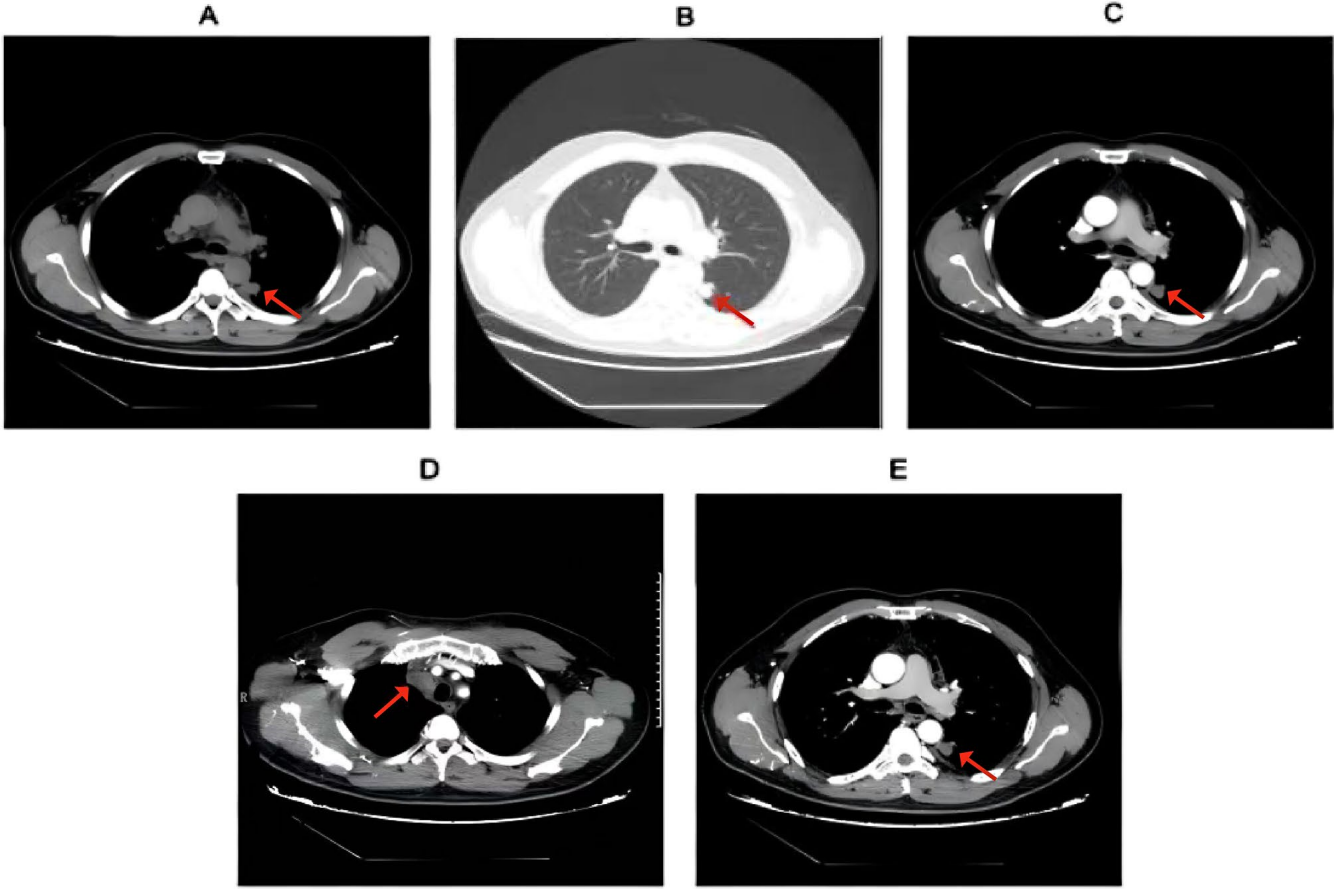
Enhanced CT, July 21, 2020. (**A**) Left lower lobe scattered ground-glass opacities, small nodules, and mild pleural thickening (suggestive of inflammatory irritation). (**B**) Bilateral thickened pulmonary markings, scattered micronodules, and patchy opacities; prominent ground-glass opacity in the left lower lobe posterior basal segment, left pleural effusion with adjacent compressive atelectasis, and bilateral peribronchiolar haziness (consistent with chronic inflammation). (**C**) Pleural thickening at the same level, notably on the right. (**D**) Superior vena cava stenosis, with abnormal enhancement of ribs and subclavian vein (suggestive of possible thrombosis). (**E**) Multiple enlarged mediastinal lymph nodes, plus calcified lymph nodes in axillary and hilar regions

On July 21, chest CT showed stable bilateral pulmonary nodules, left lower lobe chronic inflammatory lesions, persistent left pleural effusion, and new thrombosis of the superior vena cava and subclavian vein (Fig. 4).

Following multidisciplinary consultation, the patient met the clinical criteria for a diagnosis of LS (based on cervical venous thrombosis, pulmonary septic emboli, and systemic inflammation) plus right pulmonary artery embolism, pneumonia, left pleural effusion, and suspected pulmonary space-occupying lesions. He also received a new diagnosis of type 2 diabetes mellitus (due to abnormal glucose metabolism) and was initiated on insulin therapy. Treatment included anticoagulation (rivaroxaban), antimicrobial therapy with anaerobic coverage, and anti-inflammatory measures. The patient stabilized and was discharged on July 22, 2020, with a prescription for oral rivaroxaban (20 mg daily) and scheduled follow-up. During the three-week hospitalization, low molecular weight heparin (Fraxiparine-Nadroparin injection 5000 IU subcutaneously q12 h) was used, followed by oral rivaroxaban 20 mg qd after discharge, with a reduction to 10 mg qd after six months and stopping the medication after one year. At the same time, piperacillin-tazobactam was administered at a dose of 4.5 g three times a day for a course of 2 weeks.

### Stage 3: diagnosis of metastatic lung adenocarcinoma

Two weeks post-discharge (August 10, 2020), a positron emission tomography computed tomography (PET-CT) scan was performed. While it ruled out LM, it confirmed metastatic lung adenocarcinoma: a primary nodule in the left lower lobe dorsal segment, multiple metastatic mediastinal lymph nodes, persistent left pleural effusion and bone metastases. The diagnosis was pathologically confirmed by a right supraclavicular lymph node biopsy, which demonstrated metastatic adenocarcinoma.

Following the confirmation of an EGFR exon 19 deletion (19del) mutation, first-line targeted therapy with afatinib (40 mg orally once daily) was initiated in August 2020. Zoledronic acid was administered concurrently for the management of bone metastases. A subsequent chest CT scan demonstrated a partial response (PR) to this regimen. In May 2021, molecular testing revealed the emergence of a T790M resistance mutation. Accordingly, the treatment was switched in June 2021 to second-line osimertinib (80 mg orally once daily), which maintained the partial response on follow-up imaging.

### Stage 4: progression to leptomeningeal metastases

In mid-2024, the patient developed new-onset headache. Brain MRI revealed abnormal meningeal enhancement in the right frontal and occipital lobes. Cerebrospinal fluid (CSF) analysis detected EGFR 19del, TP53, and CTNNB1 mutations, confirming LM. Consequently, the treatment was adjusted to third-line therapy with furmonertinib (160 mg orally once daily).

By late 2024, the disease progressed in the central nervous system (CNS) with acquired resistance to targeted therapy. Following multidisciplinary discussion and after obtaining patient consent, combination therapy was initiated. The regimen consisted of pemetrexed (0.9 g IV, day 1), carboplatin (600 mg IV, day 1), bevacizumab (0.6 g IV, day 1) every 3 weeks (Q3W), while furmonertinib was continued. Carboplatin was subsequently discontinued, and the patient was maintained on a combination of pemetrexed, bevacizumab, and furmonertinib.

**Fig. 5:**
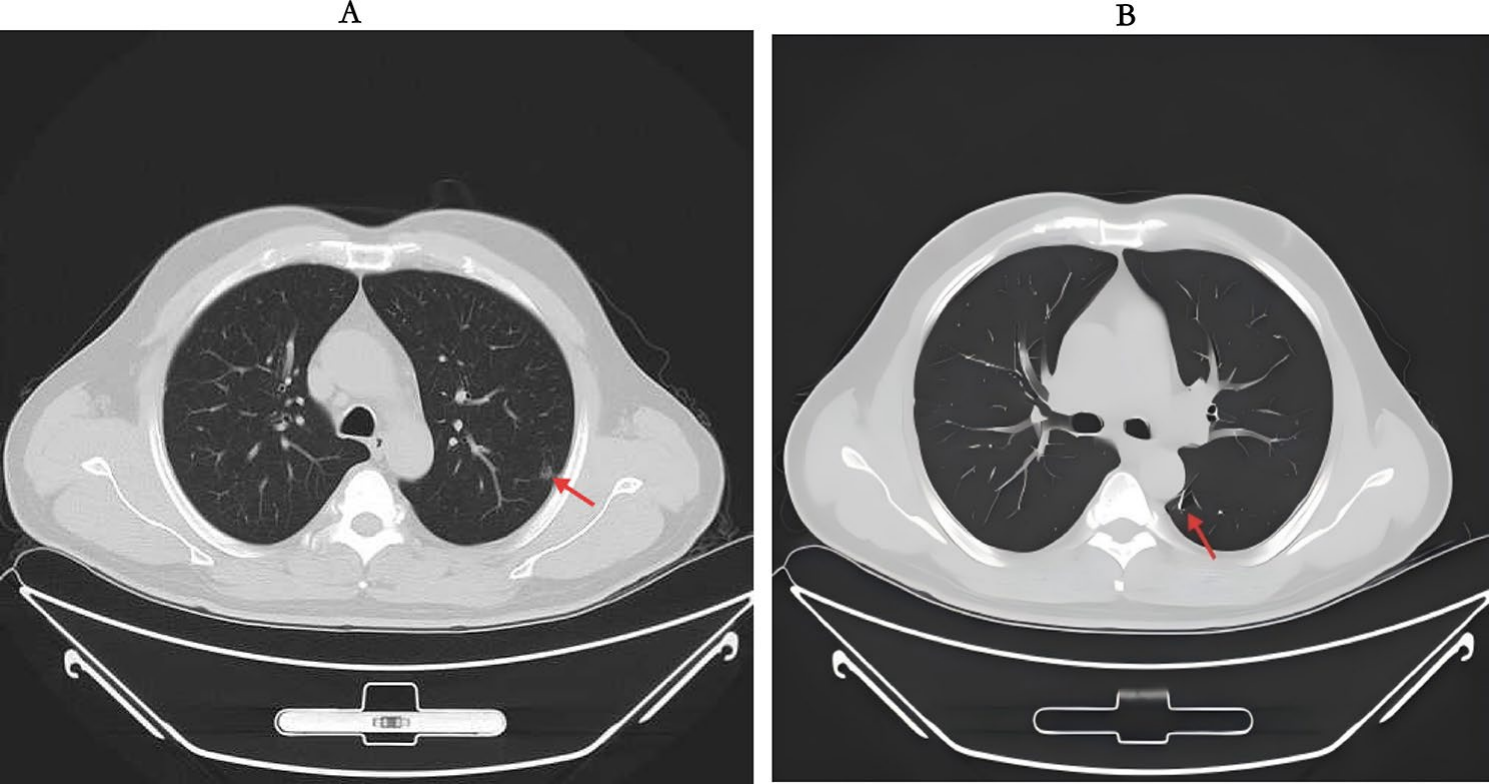
Follow-up chest CT findings on May 6, 2025. (**A**) Axial image shows an irregular subpleural nodule in the left upper lobe, multiple bilateral micronodules, and scattered enlarged mediastinal lymph nodes. (**B**) A different slice demonstrates a striped shadow with local pleural traction in the left lower lobe

In March 2025, brain contrast-enhanced MRI with T2 FLAIR revealed mild cortical swelling in the bilateral frontal lobes and parieto-occipital junction, along with abnormal signals in the left insular region suggestive of chronic ischemic changes. Patchy linear enhancements were observed in the sulci of the bilateral occipital lobes and left frontal lobe, consistent with LS. On May 6, 2025, a chest CT scan showed a slight increase in bronchial markings in both lungs, an irregular solid nodule under the pleura of the left upper lobe (Se203 Im102); multiple scattered small nodules in both lungs, some with a slight increase in density; scattered enlarged lymph nodes in the interstitial spaces (Fig. 5). A neck ultrasound examination on May 6, 2025, revealed: narrowing of the right internal jugular vein with a width of about 3 mm, and isoechoic filling was observed inside. CDFI showed no significant blood flow signal within the lumen. The imaging diagnosis suggested an old thrombus in the right internal jugular vein.

**Fig. 6:**
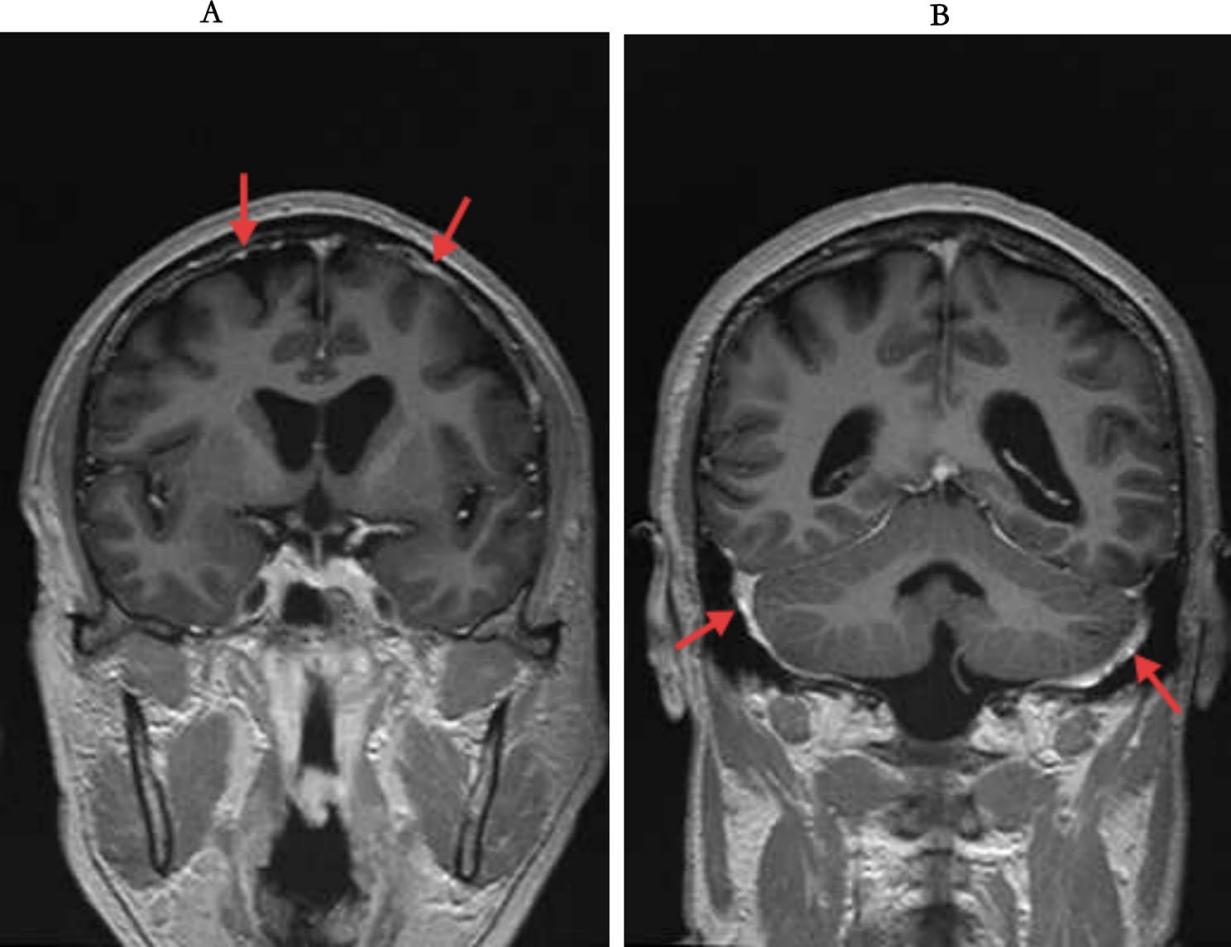
Brain MRI findings of leptomeningeal metastases on May 6, 2025. (**A**) Axial post-contrast T2-FLAIR image demonstrates characteristic linear leptomeningeal enhancement along the cerebral sulci; (**B**) Linear enhancement is also seen along the cerebellar sulci, with associated subcortical patchy hyperintensities

On the same day, brain enhancement and enhanced T2 Flair were performed (Fig. 6), which showed brain meningeal metastatic lesions on FLAIR with strip-like enhancement visible on the surfaces of both cerebral hemispheres and the cerebellum.

In addition, a cerebrospinal fluid (CSF) cytological examination was also performed, and the diagnostic result showed that adenocarcinoma cells were detected in the cerebrospinal fluid.

On May 8, 2025, the patient underwent a seventh cycle of combined chemotherapy and targeted therapy, including pemetrexed 0.9 g IV and bevacizumab 0.6 g IV on day 1 every 3 weeks (Q3W), with an increased dose of furmonertinib to 240 mg daily. Post-treatment, the patient reported occasional headaches but no significant respiratory or gastrointestinal symptoms. Subsequently, the patient was readmitted on May 29, 2025, for further evaluation and management of the malignant pulmonary neoplasm. After the exclusion of contraindications, the eighth cycle of the same combination regimen was administered during this admission. The patient was discharged in stable condition with normal vital signs, no cardiopulmonary and gastrointestinal symptoms, and no adverse events.

## Discussion

This case details the first reported instance of LS concurrent with EGFR-mutant LADC progressing to LM, a combination that expands clinical understanding of overlapping infection and malignancy. Prior case reports have documented the association between LS and underlying head and neck malignancies. Specifically, instances of LS concurrent with thyroid cancer, and malignancies of the tongue and esophagus have been reported [12–14]. Unlike prior cases of LS with underlying malignancies, this report uniquely highlights the diagnostic and therapeutic challenges of managing two rare, progressive conditions: a life-threatening infectious thrombotic disorder and a metastatic epidermal growth factor receptor EGFR-mutant lung cancer.

LS is classically defined by oropharyngeal infection-induced internal jugular vein thrombophlebitis and septic emboli, with pulmonary involvement in up to 92% of cases [15]. LS has not been previously associated with LADC or LM. The patient’s atypical initial presentation (chest pain, bilateral pulmonary inflammation without oropharyngeal symptoms) delayed LS diagnosis until cervical vein thrombosis and pulmonary embolism developed. Concurrent type 2 diabetes—an immune-suppressive condition—likely exacerbated infection severity and masked LS’s typical prodrome [16]. A critical clinical lesson emerges: in immunocompromised LS patients, persistent pulmonary nodules or lymphadenopathy post-infection control warrants proactive malignancy screening (e.g., PET-CT), even in the absence of overt cancer symptoms.

Prior studies have linked *Fusobacterium necrophorum* (the primary pathogen in LS) to colorectal cancer progression via oncogenic pathway activation (e.g., β-catenin) and immune suppression [17]. Similarly, chronic oropharyngeal infections are emerging as promoters of tumorigenesis in lung cancer via local inflammation and epithelial-mesenchymal transition [18]. In this case, the temporal relationship, LS preceding overt LADC metastasis, raises the hypothesis that infection-induced inflammation may have accelerated LADC progression or masked its early signs. Conversely, advanced malignancies impair innate immunity (e.g., via neutropenia or cytokine dysregulation), increasing susceptibility to severe bacterial infections [19]. This “chicken-or-egg” dynamic highlights the need for future studies exploring microbiome-tumor interactions in lung cancer patients with rare infections like LS.

The successful stabilization of this patient relied on three core pillars of care, all of which offer broader clinical lessons. The role of anticoagulation in LS remains controversial, with guidelines recommending it only for antibiotic failure or progressive thrombosis [20–21]. However, given the patient’s severe cervical swelling, pulmonary arterial embolism, and elevated D-dimer (4.83 mg/L), the MDT prioritized early rivaroxaban initiation (20 mg daily), a decision that prevented thrombus extension without increasing bleeding risk.

Upon LM diagnosis (mid-2024), the MDT integrated third-generation EGFR-TKI (furmonertinib, escalated to 240 mg daily) with pemetrexed-bevacizumab chemotherapy, leveraging furmonertinib’s favorable CNS penetration [22] and anti-angiogenics’ ability to disrupt LM microenvironment [23]. This combination achieved stable disease until May 2025, demonstrating the value of tailored, cross-specialty therapy.

The patient’s treatment trajectory (afatinib to osimertinib to furmonertinib) aligns with current guidelines for EGFR-mutant LADC, but LM progression required dose escalation, an adjustment supported by phase II data showing improved CSF penetration with high-dose furmonertinib (240 mg vs. 160 mg) [24]. This underscores the need for CNS-focused dose optimization in LM, even for third-generation TKIs.

A key lesson is the importance of extended, cross-system follow-up for patients with LS and malignancy. After LS resolution (2020), PET-CT identified LADC, highlighting that post-infection imaging should not be limited to infection-related sites. Annual brain MRI (initiated in 2021) detected LM early (mid-2024), enabling timely treatment adjustment. For EGFR-mutant LADC patients, current guidelines recommend brain MRI every 6–12 months [25]; this case supports adherence to this schedule, even in the absence of neurological symptoms.

## Conclusion

As the first documented case of Lemierre syndrome coinciding with EGFR-mutant lung adenocarcinoma and its progression to leptomeningeal metastasis, this report provides critical insights for managing such rare comorbidities. This case offers critical clinical lessons: pulmonary lesions that remain unresolved after treatment for LS should warrant a high index of suspicion and prompt investigation for occult malignancy. Furthermore, it underscores the importance of routine CNS surveillance in EGFR-mutant LADC, particularly with a history of severe infection, to enable timely intervention for LM. Ultimately, the patient’s outcome demonstrates that success in these complex scenarios hinges on multidisciplinary collaboration to balance and tailor therapies for both the life-threatening infection and the advanced cancer.

## Electronic Supplementary Material

Below is the link to the electronic supplementary material.

Supplementary Material 1

Supplementary Material 2

## Data Availability

No additional data.

## Abbreviations

LS: Lemierre syndrome
LC: Lung cancer
CT: Chest computed tomography
CRP: C-reactive protein
AFP: Alpha-fetoprotein
CEA: Carcinoembryonic antigen
CSPINE: Cervical spine
CNS: Central nervous system
PR: Partial response
CSF: Cerebrospinal fluid
ECG: Electrocardiography
US: Ultrasonography
CK: Creatine kinase
MDT: Multidisciplinary team
TKIs: tyrosine kinase inhibitors
