# Supplementary material for: Leptomeningeal metastasis from lung adenocarcinoma associated with Lemierre syndrome in a middle-aged man: a case report and review of the literature
Source: BMC Infect Dis. 2026 Jan 14;26:304. doi: 10.1186/s12879-025-12359-3 (PMC12888586; doi:10.1186/s12879-025-12359-3)
Supplement: Supplementary file 1 — Supplementary Material 1 [file 12879_2025_12359_MOESM1_ESM.pdf]

Informed Consent Form for Medical Case Reports

Patient Statement

After being fully informed by attending physician Yang Xiaoxia, I, Liu Hongqi, hereby expressly acknowledge and confirm the following matters:___

1. Publishing authorization:

The license authorizes the publication of de-identified medical cases in academic journals, including print publications, digital platforms, and materials from related academic conferences.

According to the "Privacy Desensitization Standards" in the appendix, authors must remove 18 categories of directly identifiable information such as ID numbers and addresses, but it should be understood that rare cases with distinctive features or special treatment details may still be indirectly identified by industry professionals.

The authors agreed to submit the original image data to the journal for peer review.

2. Rights reserved:

3. The withdrawal period is from the date of signing this document to the deadline for final layout of the journal.

4. Withdrawal procedure: The applicant or their legal representative must submit a written withdrawal application to the hospitals research management department with valid identification documents.

5. Restriction clause: Articles indexed in PubMed cannot be retracted.

6. Risk Warning:

The potential impacts include academic literature citation, news media reprint, and pharmaceutical industry research reference.

7. Journal safeguard measures:

The contact information of patients shall be kept strictly confidential.

The images of cases were blurred in the eyes.

Data on cases must not be sold to commercial entities.

8. Legal basis

According to Article 1219 of the Civil Code and the Measures for the Ethical Review of Biomedical Research Involving Human Subjects:

The attending physician has fulfilled the duty of informed consent through: ☐ face-to-face oral explanation ☐ remote video consultation ☐ written and visual materials

☐ For minors or persons without civil capacity, the document must be signed by a legal representative and notarized by the hospitals ethics committee for record.

Sign and take effect

Patient Signature: Date:

(Information below is only for medical institutions to keep two copies. 8.) 4

ID number: 320825197803031730 Emergency contact: Sheng Xiuhua (spouse) Tel:

13575452973

Legal Representative Attachment (if applicable):

☐ Copy of guardians ID card ☐ Household registration book and kinship certificate ☐ Guardian documents confirmed by attending physician:

I confirm that the examinee/attendant has fully understood the document content and that there was no improper interference during the signing process.
Signature: Xiang ID: L042 Date: 5 8 ______
