# Supplementary material for: Leptomeningeal metastasis from lung adenocarcinoma associated with Lemierre syndrome in a middle-aged man: a case report and review of the literature
Source: BMC Infect Dis. 2026 Jan 14;26:304. doi: 10.1186/s12879-025-12359-3 (PMC12888586; doi:10.1186/s12879-025-12359-3)

**Supporting Information for**

**Letter to the Editor**

**Leptomeningeal metastasis from lung adenocarcinoma associated with Lemierre syndrome in a middle-aged man: a case report and review of the literature**

Xiaoxia Yang^1^, Jingang Han^1*^, Zhi Yu^1^, Jianhua Nian^1^, Jie Chen^1^

^1^Department of Vascular Surgery, The Second Affiliated Hospital of Zhejiang Chinese Medical University, Hangzhou, China.

# *Corresponding Author

hanjingang1976@sohu.com

**Supporting Tables, Figures**

Table 1 The corresponding timeline of diagnoses and management .

| **Time** | **Diagnosis** | **Investigation** | **Management** |
| --- | --- | --- | --- |
| July 1, 2020 | 42-year-old male presented with acute right-sided chest pain | Chest CT:  Bilateral pulmonary inflammation, multiple scattered nodules/micronodules and mediastinal lymphadenopathy | suggestive of septic emboli |
| July 13, 2020 | Definite diagnosis of LS | Neck Enhanced MRI:  Right internal/external jugular & subclavian vein thrombosis confirmed. | LS treatment (anticoagulation, broad-spectrum antibiotics,anti-inflammatory agents) initiated |
| During Follow-up | Developed chest pain;  Diagnosis of LADC confirmed | Pathology:  Confirmed LADC. | LADC-specific therapy started, combining targeted agents and chemotherapy |
| 2024-2025 | Reported persistent headache; Diagnosis of LM confirmed | Brain Enhanced CT: LADC leptomeningeal metastasis confirmed. . | Treatment intensified with dose adjustment of the EGFR TKI, Osimertinib |

**Fig. 1** Chest CT scan on July 1, 2020.


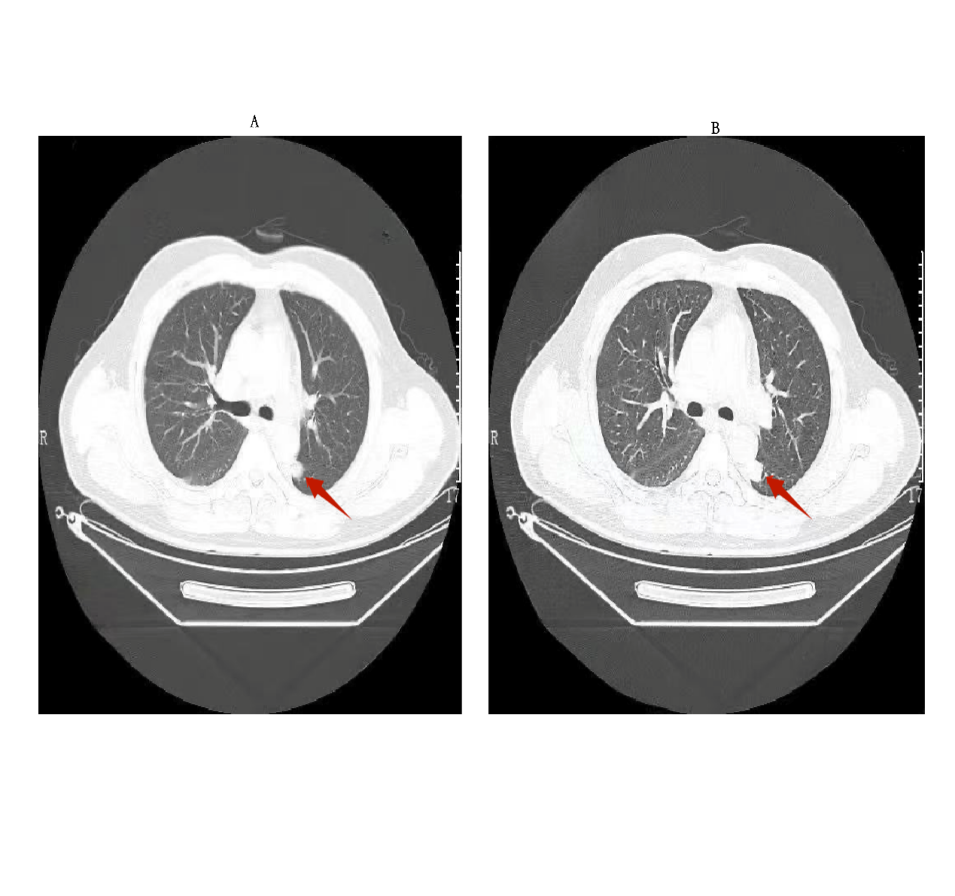


**Fig. 2** Cervical contrast-enhanced MRI on July 13, 2020.


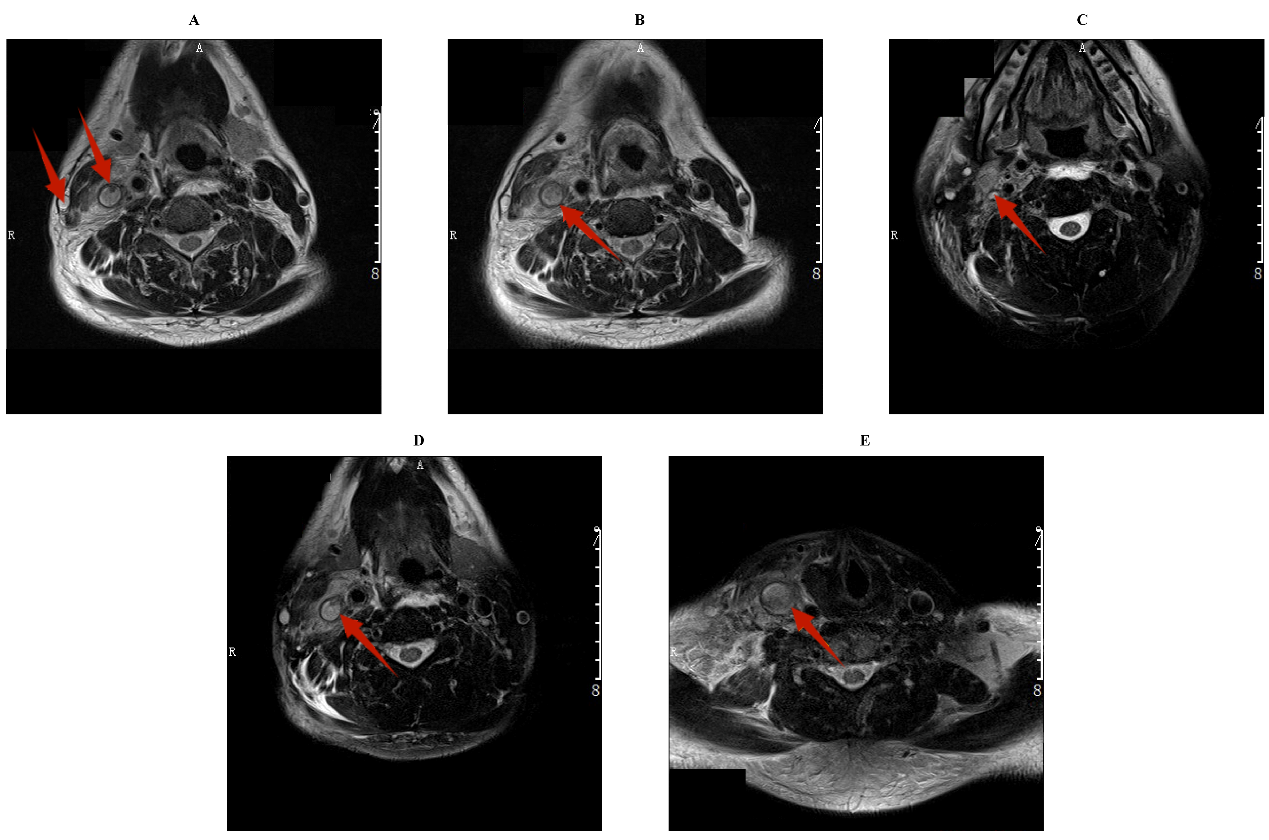


Fig. 3 Computed tomography pulmonary angiogram (CTPA) on July 17, 2020.


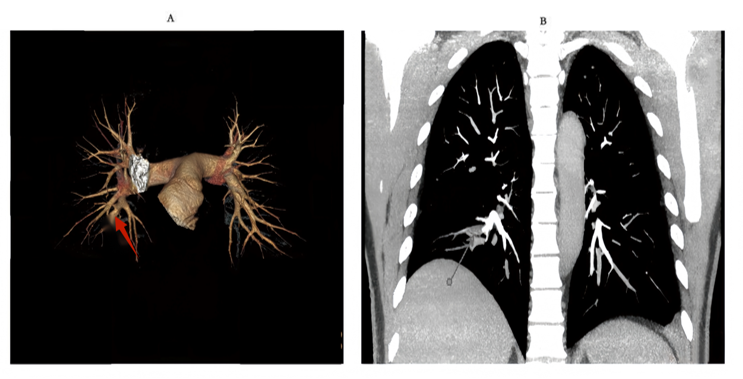


Fig. 4 Enhanced CT on July 21, 2020.


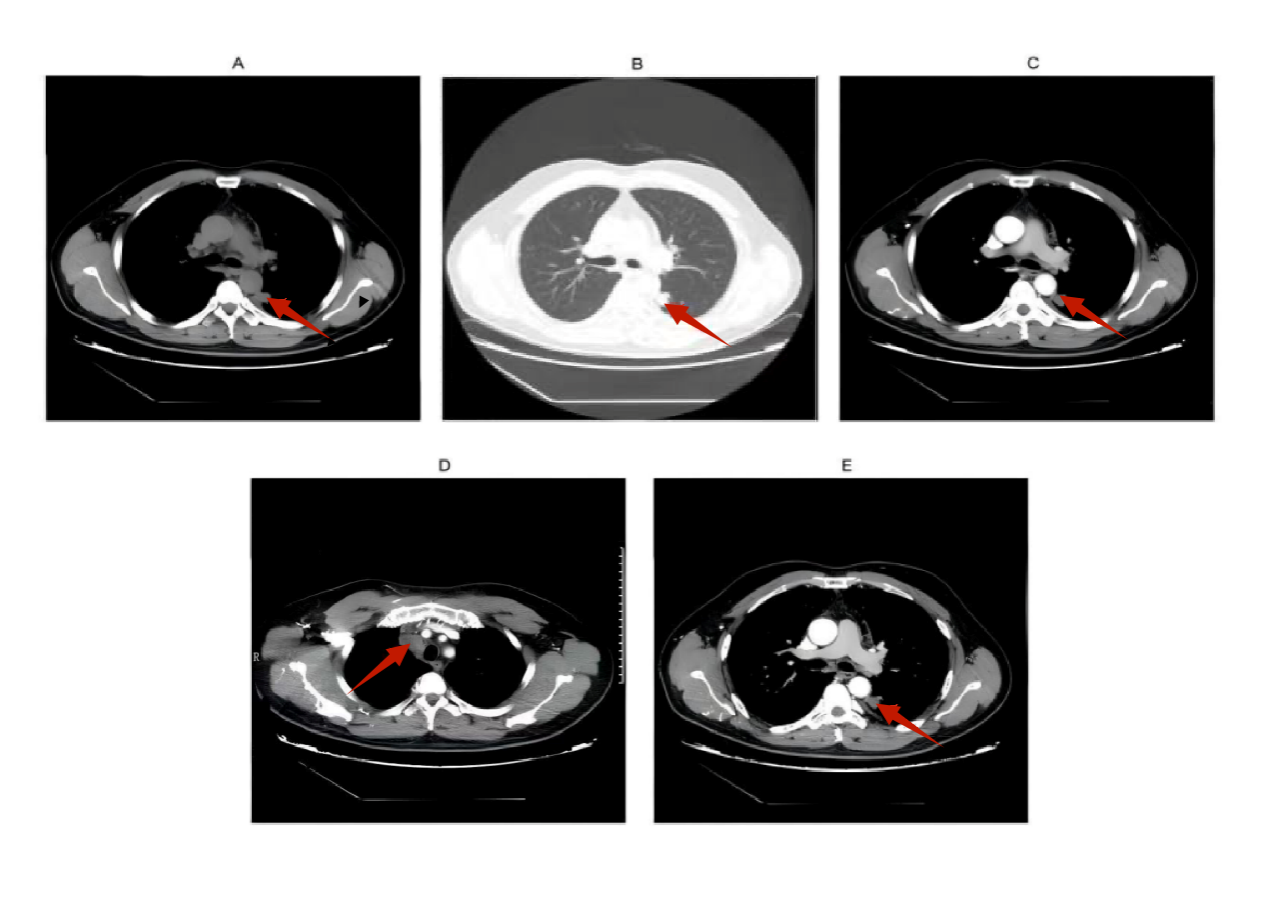


**Fig. 5** Follow-up chest CT findings on May 6, 2025.


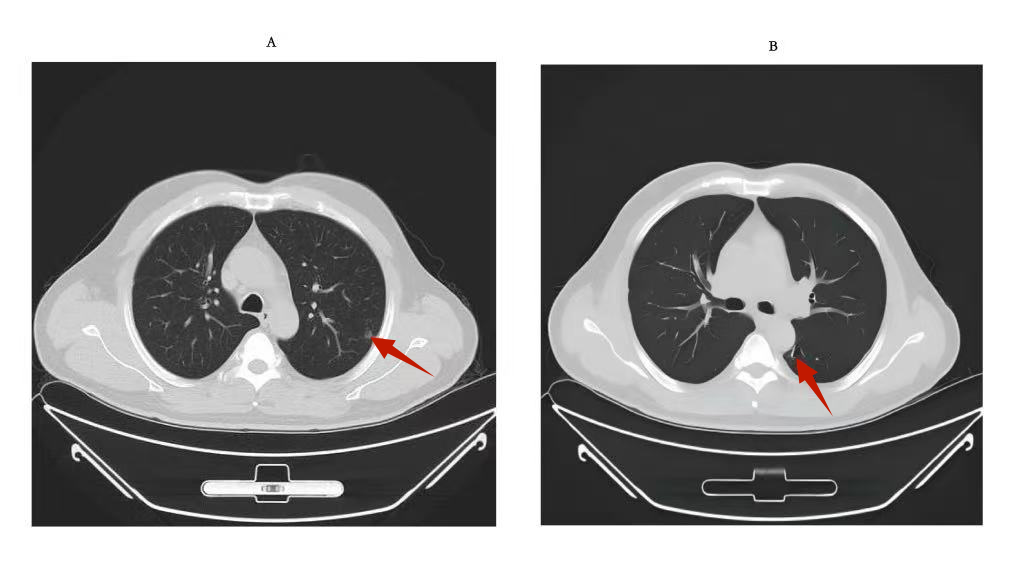


**Fig. 6** Brain MRI findings of leptomeningeal metastases on May 6, 2025.


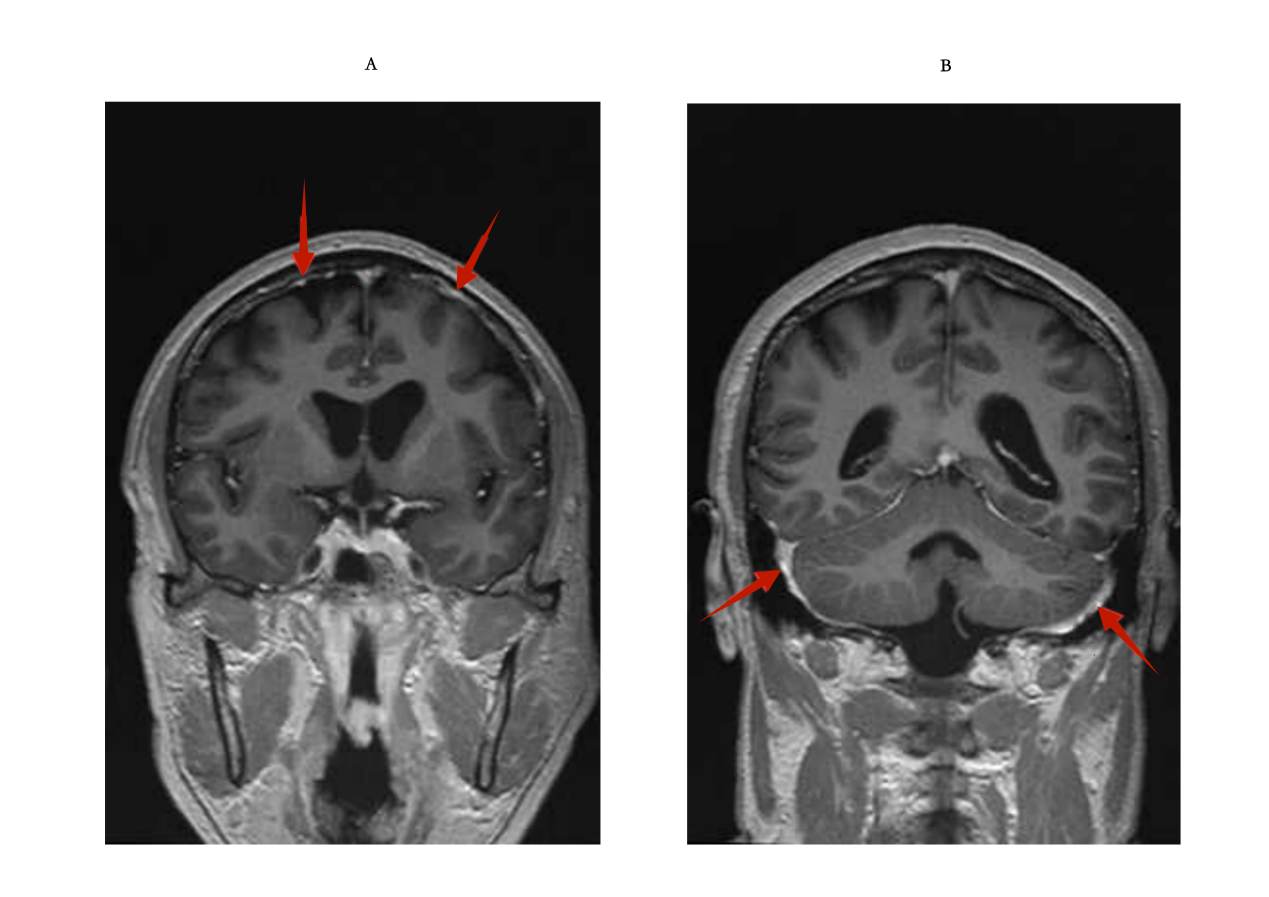

Supplement: Supplementary file 2 — Supplementary Material 2 [file 12879_2025_12359_MOESM2_ESM.docx]
